# Medical Applications of Skin Tissue Dielectric Constant Measurements

**DOI:** 10.7759/cureus.50531

**Published:** 2023-12-14

**Authors:** Harvey N Mayrovitz

**Affiliations:** 1 Medical Education, Nova Southeastern University Dr. Kiran C. Patel College of Allopathic Medicine, Fort Lauderdale, USA

**Keywords:** wounded skin, diabetic skin, lymphedema measurement, breast cancer related lymphedema, bcrl, breast edema, edema, lymphedema, permittivity, tissue dielectric constant

## Abstract

Tissue dielectric constant (TDC) values assess certain skin properties that are dependent on multiple factors but mainly on the relative amount of water content within a locally measured tissue volume. Because of the non-invasive nature of these measurements and their ease of use, the method has been widely used in various medically related applications. The goal of this paper was to review and describe the uses and findings of such TDC measurements, considering and including the wide array of medical applications. The review is in part based on information derived from an analysis of published material obtained via literature searches of four major electronic databases and, in part, based on the author’s experience with the TDC measurement methods and their various applications and his professional experiences. The databases searched were PubMed, Web of Science, EMBASE, and CINAHL Complete. Based on the initial search criteria, a total of 1257 titles were identified. After removing duplicates and filtering according to relevancy, 160 remained for detailed further review. In some cases, the bibliography of these retrieved articles provided additional sources. The findings demonstrate multiple research and medical uses and applications of TDC measurements, focusing on detecting and quantifying localized edema and lymphedema in multiple target sites. These include the upper and lower extremities, breasts, and trunk as regions involved in medical conditions causing lymphedema. In addition, the findings suggest that TDC evaluations are a convenient, non-invasive method to study and evaluate other conditions impacting skin, including diabetes mellitus and skin wounds or ulcers. Its ability to detect aspects of tissue changes simply and rapidly at almost any anatomical location makes it a useful tool for investigating multiple dermatological conditions and their treatment as future applications of this method.

## Introduction and background

**Figure 1:**
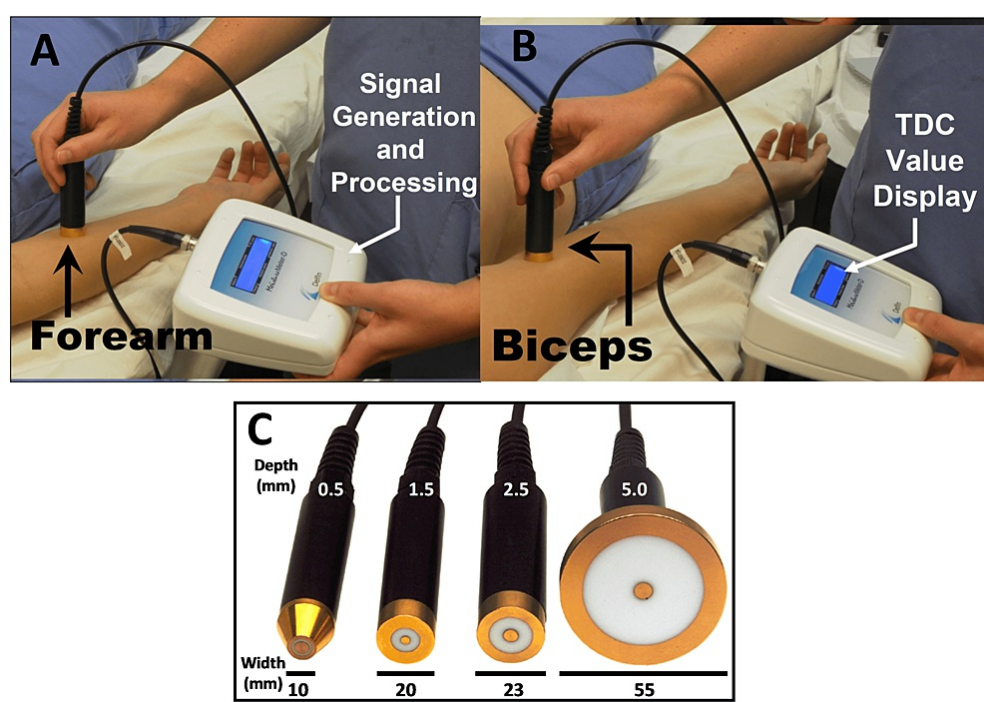
TDC probes and measurement applications on the arm (A) and (B) show measurements on the forearm and biceps using the 2.5 mm depth probe. Part (C) shows the probes available, their outside diameter, and their effective measurement depths. The probes are connected to a control box that generates a 300 MH signal and processes the reflected signal that is used to determine the TDC value that is displayed on the display. The figure is courtesy of Dr. HN Mayrovitz.

The term tissue dielectric constant (TDC) was originally introduced in 2007 to refer to the permittivity of biological tissue as measured at a frequency of 300 MHz [1]. At that time, the aim was to evaluate the skin-to-fat tissue of the arm to characterize the relative amount of water in localized tissue to assess breast cancer-related lymphedema (BCRL). However, measurements of the dielectric constant of biological materials had long predated that time. Pioneering work in the application of such measurements was done by Schwan et al. [2-5], who built upon and extended the work of Cole et al. related to cell suspensions [6,7]. The use of radio and microwave frequencies combined with transmission line principles and associated mathematics led to the development of methods to measure dielectric constant values of tissues in vitro from various organs [8-12]. Measurements were made in vivo in a single subject [13], but for the most part, these tissue measurements were confined to the laboratory given the equipment needed. However, based on analyses and engineering design efforts [14-16], a device in the form of a portable open-ended transmission line emerged [17]. A commercialized version of the measurement system was developed that had four different-sized probes with diameters ranging from 10 to 55 mm and connected to a control box that generated a 300 MHz signal, as pictured in Figure 1.

**Figure 2:**
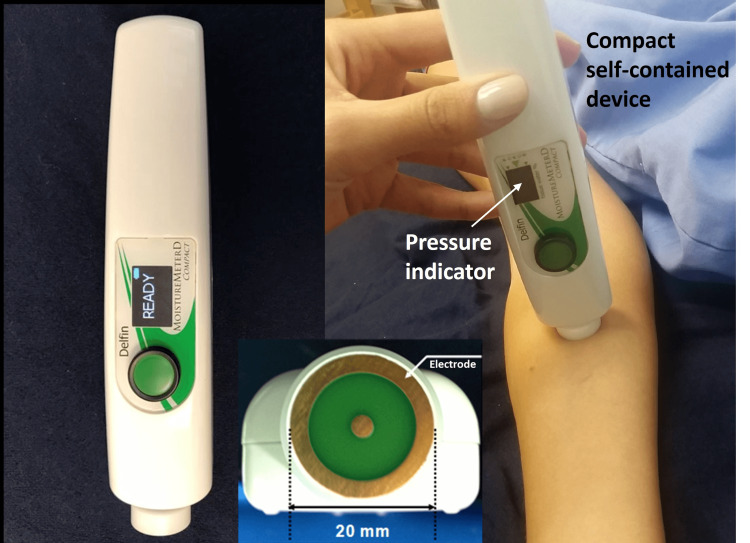
Compact version of the TDC measurement device The outside diameter of the outer electrode is shown as 20 mm. The figure illustrates a measurement of the forearm with the compact device. The figure is courtesy of Dr. HN Mayrovitz.

The 300 MHz signal was sent through the probe, which functioned as an open-ended transmission line. As a consequence, the reflected wave could be analyzed within the software of the control box to calculate the effective dielectric constant through well-established methods [9,14,18]. With further developments in technology, these processes were all integrated into a compact device, shown in Figure 2, that had an effective penetration depth of between 2.0 and 2.5 mm [19].

These developments in technology and capability allowed the utilization of TDC values for the assessment of skin tissue properties that were revealed by the dielectric constant of the tissue volume interrogated by the incident electromagnetic wave. One of these properties relates to the amount of water within the measured tissue volume since the TDC value is strongly dependent on tissue water content [20]. As a consequence, measurements of TDC provide a convenient method to assess tissue water and its change. Capitalizing on this property, some of the earliest medical applications targeted conditions in which tissue water and its change were of clinical interest, such as cerebral edema [21-23] or lung edema [24] which were studied in experimental animals. Applications that used probes similar in function to those shown in Figure 1 and applied to humans in 2003 targeted changes in skin-to-fat water associated with cardiac surgery in a group of 29 patients [25]. Subsequently, TDC measurement use has expanded to a variety of medical applications. One of these is related to assessing upper extremity lymphedema, which is a complication of breast cancer treatment referred to as BCRL. This condition has an incidence that ranges from 20 to 60% [26-30] and is evaluated with various methods to detect its presence and track its progression. These include arm circumference measurements [31-34], arm volume measurements [35-39], and the determination of the electrical impedance of the arms [40-44]. This same type of measurement can be applied when the edematous or lymphedematous condition is in the lower extremities [45-48]. However, when it comes to measuring localized edema or lymphedema, either at specific locations on the limbs or anywhere on the body where lymphedema detection or tracking is indicated, there are limited methods available. One such method, which is the main subject of this paper, is the measurement of TDC. Thus, the specific aim herein was to review and describe the uses and findings of TDC measurements, considering and including their wide array of medical applications.

## Review

Methods

This review is in part based on information derived from an analysis of published material obtained via literature searches of four major electronic databases and, in part, based on the author’s experience with the TDC measurement methods and their various applications, his professional experiences, and the original material of the author. The databases searched were PubMed, Web of Science, EMBASE, and CINAHL Complete. The primary search term strategy for each of these was as follows: The term “dielectric constant” was searched in combination with a logical AND for the term’s “skin” or “edema” or “lymphedema” as (dielectric constant) AND (skin OR edema OR lymphedema). Using this strategy, a total of 1257 titles were identified. Duplicate titles from the four databases were eliminated, and the retrieved titles were screened for potential relevance via an abstract review for further clarifications if warranted. This review and filtering process reduced the likely relevant articles to 160. The present author was a primary or co-author on about half of these (59), and the remainder were retrieved if not already available and reviewed. In some cases, the bibliography of the retrieved articles provided additional sources. Supplemental searches were done as needed. The manuscript is organized as follows: The first part describes general aspects of TDC applications by considering many of the main factors that impact their value and interpretation. These include factors such as the number of measurements per site, variations by anatomical location, depth of the measurement, subject age, gender, or race, and even the time of day of the measurement. This is followed by a review of its many medical applications.

General aspects

Each available device has pros and cons. The multiprobe set (Figure 1) can measure to varying depths (0.5 mm to 5.0 mm) [49] but is less mobile than the compact version (Figure 2). The compact device also has a pressure sensing process to help increase repeatability but offers measurements only to a single depth [50].

Number of TDC Measurements per Site

The measured TDC value is localized in that it reflects the value of the tissue volume roughly equal to the product of the probed diameter and its effective measurement depth. Because the TDC value largely depends on tissue water content, it is useful in the assessment of edema, lymphedema, and its change. As with any such measurement, there is a need to balance the number of measurements per site with the time required for multiple measurements. This aspect has been studied [51,52].

In one study, similar TDC values were found based on a single measurement or the average of triplicate measurements in healthy and lymphedematous arms. In that study, ventral forearms were measured bilaterally to a depth of 2.5 mm in 10 women with unilateral arm lymphedema. The 95% confidence interval for differences between single and averaged values was found to be less than ± 1 TDC unit for both arms [51]. Another study measured the anterior forearms of 20 healthy women to 0.5, 1.5, 2.5, and 5.0 mm depths and also measured the lateral thorax bilaterally on 10 women with BCRL to a depth of 2.5 mm. The 95% confidence interval for differences between single and averaged TDC values was less than ± 1 TDC unit, and the limits of agreement between methods were less than ± 2.5 TDC units (± 6.5%) for each condition, site, and depth measured. If this level of agreement is acceptable, suitable clinical assessments can be made using a single TDC measurement.

Extensions to other anatomical locations were carried out on a group of 60 female subjects [53]. TDC was measured bilaterally in triplicate at the forearm, hand palm, lateral calf, medial calf, and foot dorsum. Single, duplicate, and triplicate values were compared for absolute TDC values and inter-side ratios. Differences between single and multiple measurement averages were anatomical site-dependent, with the smallest coefficient of variation (2.19%) at the forearm and the largest at the lateral calf (4.59%). Thus, if clinical time is of major concern, useful TDC data may be obtained in upper limbs by a single TDC measurement per site, but lower extremity skin assessments are best with duplicate and preferably triplicate measurements.

Variations in TDC Values by Anatomical Location

This issue has been studied at various sites on the forearm, different upper arm sites, and lower extremities [49,54-56]. In a study of 30 healthy women, TDC was measured on the anterior forearm midline and also 1.2 cm medial and lateral to the midline at sites 4, 8, and 12 cm distal to the antecubital crease [54]. The midline and medial TDC values increased progressively from 4 to 8 to 12 cm sites (p<0.001). At a depth of 2.5mm, TDC values increased, with a maximum difference of 8.2 ± 10.6%. For all sites, TDC values were lower (p<0.001) with increasing depths. Thus, TDC increases from proximal to distal sites and should be considered since such differences are important when evaluating patients with arm lymphedema [56]. Handedness does not appear to have an impact on measured TDC values [57].

Other information on the variability of TDC by location emerged from a study of 36 women who were followed for 78 weeks after their breast cancer surgery [56]. TDC and arm girth were measured bilaterally at multiple arm sites on multiple occasions. An inverse correlation was found between TDC and girth (p<0.001), but TDC values decreased with distance from the wrist (p<0.001). Differences in TDC values between anterior and medial sites at the same longitudinal position have also been investigated [58]. Forearm TDC was measured to multiple depths in 40 women, with no statistical differences between anterior and medial sites found. In that study, a moderate correlation was found between the total body water percentage and the TDC value, which was greatest when measured to a depth of 5 mm.

Variations in TDC Values by Measurement Depth

TDC values depend on free and bound water in the measured tissue volume; thus, tissues with more fat hold less water, and the TDC value is lower. Thus, in anatomical areas with more subcutaneous fat, TDC values tend to be lower, especially when measurements are made at deeper levels. This finding has been experimentally demonstrated [59-61]. In one of these studies, the anterior forearm TDC was determined bilaterally at depths of 0.5, 1.5, 2.5, and 5.0 mm in 40 healthy women with BMI values within the normal range. This determination was then compared to the subjects' total body water percentage (TBW%) and arm percentage fat (AF%) [62]. TDC was found to directly correlate with TBW% (r=0.512, p=0.001) and inversely correlate with AF% (r= -0.494, p<0.001). The correlations were found to be greatest at the deepest measurement depth. When TDC was measured in overweight or obese women, a similar depth dependence was obtained for measurements made on the forearms and biceps [63]. TDC differences between women who were overweight and obese were not apparent, possibly due to a threshold for TDC dependence on body fat percentage.

In contrast to the decreasing TDC values with increasing measurement depth obtained in the arm or leg, at some anatomical sites, the pattern may be different, as was demonstrated via measurements made on the hand palm, thenar eminence, great toe, and foot dorsum to depths of 0.5, 1.5, and 2.5 mm [55]. TDC values decreased with increasing depth at the hand palm, thenar eminence, and great toe, but there was essentially no change on the hand and foot dorsum. The depth to measure depends on the measurement goal, considering that some processes start deeper in tissue and others are shallower.

Variations in TDC Values by Age

The age factor was investigated in a group of 69 women in whom TDC was measured to depths of 0.5, 1.5, 2.5, and 5.0 mm on both forearms [64]. The women evaluated had a broad age range of 22-82 years and a BMI of 18.7-46.1 kg/m2. TDC values decreased with increasing depth (33.7 ± 5.8 at 0.5 mm to 21.8 ± 3.7 at 5.0 mm), but at all depths, the inter-arm ratios did not differ and ranged from 1.025 ± 0.081 at 0.5 mm to 1.017 ± 0.097 at 5.0 mm. TDC values at a depth of 0.5 mm and 1.5 mm increased with increasing age, but there was no age dependence of TDC values at 2.45 or 5.0 mm. Thus, TDC values are affected differentially by age in a depth-dependent way.

More information about the age factor emerged from a study of 200 women in which 50 women had TDC measurements made bilaterally on anterior forearms at either 0.5, 1.5, 2.5, or 5.0 mm depths [65]. The age factor was studied by comparing subjects ≤40 years with subjects ≥60 years. TDC values at 0.5 and 1.5 mm were reported to be greater for older women (p<0.001). Similar but less dramatic age-related changes were uncovered in 60 males in whom TDC was measured bilaterally to the same multi-depths on the forearm [66]. There were three groups of 20 each compared: 24.0 ± 0.9, 40.0 ± 12.9, and 71.0 ± 8.0 years. Except at a 0.5 mm depth, there was no statistical TDC difference among these groups. Taken together, the female and male results may be due to skin water shifting from bound to more mobile with increasing age. Similar results were reported for a group of 60 women in whom an age-related increase in TDC was found only to a depth of 0.5 mm [67]. Age-related changes impact long-term longitudinal tracking studies and also shape what reference values are in relation to a patient’s age.

Variations in TDC Values by Gender

The potential gender factor was investigated in a study of 30 men and 30 women in whom TDC was measured on their forearms, forehead, and cheeks to a depth of 1.5 mm [68]. TDC values had a wide range from 39.6 ± 2.9 at the forehead of the male group to 28.2 ± 2.4 at the forearm of the female group. TDC values were significantly different (p<0.001) among each site in the order forehead > cheek > forearm. Male TDC values were greater than those measured in females at corresponding anatomical sites (p<0.01), with differences ranging from 5.6% at the forehead to 11.3% at the forearm. The gender factor was consistent with later findings, in which 32 male and 32 female subjects were evaluated [19]. In this study, the device used was the compact version with a fixed measurement depth of between 2.0 and 2.5 mm. TDC data was obtained bilaterally on forearms and biceps, with the result that female values were significantly less than those obtained from males at similar anatomical sites. In a subsequent investigation of forearm TDC values in 280 young adult subjects per gender, the greater TDC values for males vs. females were confirmed for all depths of 0.5, 1.5, 2.5, and 5.0 mm [60]. Male-female percentage differences ranged from 14.8% to 22.0%. The overall gender-related findings suggest that gender differences should be considered in any study in which men and women are included in a common study population with respect to experimental design and data interpretation. This is especially true if absolute TDC values are of interest rather than changes in TDC on the same subject subsequent to an intervention.

Variations in TDC Values by Race

Potential differences in TDC among persons of different races or ethnicities were investigated in 100 persons with 20 subjects each self-identifying as Caucasian, African-American, Asian Indian, Asian, or Hispanic [69]. TDC was measured at a depth of 1.5 mm on the forearms. For females (10 per group), analysis of variance among races indicated an overall significant difference among races (p<0.01), with the difference mainly due to the larger TDC value of Caucasians vs. Asian-Indians (p<0.05). Also, TDC values for Asian and Asian-Indian groups were lower than for Caucasians. Males did not show a difference among races at the forearm but did have an apparent race-related dependence at other sites, including the chest and foot. Follow-up analyses indicated the chest difference was due to a TDC value of African Americans that was larger than either Caucasians or Asian-Indians (p<0.01). Contrastingly, differences at the ankle were due to a larger TDC value in Caucasians with respect to all other groups (p<0.01). Thus, TDC dependence on race is a factor that should be considered in assessing skin hydration comparisons that include different races. However, despite these variations among races, no such differences were found between inter-arm TDC ratios, and this ratio remains a robust indicator of unilateral tissue water changes.

Variations of TDC Values by Time of Day

Since TDC measurements may be made at variable times during the day, knowledge of potential variations in their value is useful, especially in longitudinal assessments of patients. To investigate this factor, TDC was measured multiple times during the day in a group of 12 young adult females [70]. Measurements were made at one-hour increments starting at 0800 and ending at 2000 hours at four anatomical sites: the face just below the eye, mid-cheek, anterior forearm, and medial calf. Results indicated that TDC at the eye, cheek, and forearm decreased during the day with morning-to-night changes of 11.2 ± 8.3%, 6.8 ± 5.7%, and 5.6 ± 6.0%, respectively. Contrastingly, calf TDC values progressively increased with morning-to-night increases of 9.3 ± 10.7%. Thus, when absolute values of TDC are of relevance, the time of day of measurement may be important. However, when inter-limb ratios are used to assess the water status, the time of day is of much less importance [71].

Reliability Considerations

To gain insight into the reliability of these TDC measurements, a test-retest investigation was done in which TDC was measured in 40 healthy persons by two measures [72]. The measurements were made on multiple anatomical sites and were able to estimate the minimum detectable change (MDC) of TDC measurements and the interclass coefficients (ICC). The MDC was between two and nine TDC units, with inter-side ratios ranging from 5.3% to 8.0%. ICC values were between 0.765 and 0.982. These variations depended on the measurement depth and the body fat and water percentage. These findings were amplified in a study of 30 women who had confirmed BCRL and in whom intra- and inter-rater reliability was determined with TDC measurements at multiple sites [73]. ICC values were between 0.648 and 0.947 for intra-rater measurements and between 0.606 and 0.941 for inter-rater measurements. The range was dependent on the anatomical sites measured.

TDC applications in relation to BCRL

Upper Extremity Lymphedema

A study that focused on TDC measurements in relation to BCRL was reported in 2007 [1]. TDC was measured bilaterally to multiple depths in the forearms of 18 healthy women who served as controls and in 15 women who had been diagnosed with unilateral BCRL (patients). TDC in the lymphedematous arms of the patients was greater than in their contralateral arms (p<0.001). At a measurement depth of 2.5 mm, inter-arm TDC ratios for patients were 1.64 ± 0.30 vs. 1.04 ± 0.04 for controls (p<0.001). No patient’s TDC ratio was ≤1.2, and no control’s TDC ratio was ≥1.2.

Further work evaluated 90 women, including 30 women with unilateral BCRL, 30 women with breast cancer awaiting surgical treatment, and 30 healthy controls [74]. TDC was measured bilaterally to determine inter-arm TDC threshold ratios. TDC values did not differ between control groups, but the women with BCRL had higher values. Inter-arm ratios for the 60 women without lymphedema were 1.006 ± 0.085 and were significantly less than for the women with BCRL, who had an inter-arm TDC ratio of 1.583 ± 0.292. An at-risk/contralateral TDC ratio of 1.26 was suggested as a possible threshold for detecting preclinical or latent lymphedema.

To further study the utility of these inter-arm TDC thresholds for detecting and tracking BCRL, TDC values were obtained in 240 women [75]. A threshold was defined in this case as the mean value plus three standard deviations (SD) above the mean. Based on these measurements, a threshold TDC ratio of 1.2 was suggested. Further investigation of the use of inter-side ratios for early detection was undertaken by measuring 100 women who had been treated with breast surgery, axillary dissection, and radiotherapy [76]. It was determined that 18.4% of early BCRL were detectable only with this method because early events started localizing superficially. Also, of the 38% of patients who had clinical lymphedema, the TDC method was reported to have a sensitivity of 65.8% and a specificity of 83.9%.

Further studies were done on 207 women, of whom 104 had experienced breast surgery for breast cancer and 103 had breast cancer but were awaiting their surgery [77]. TDC was measured to 2.5 mm bilaterally in forearms and biceps, and inter-arm ratios were determined. An inter-arm TDC threshold for early detection of pre-clinical unilateral lymphedema of 1.3 for the forearm and 1.45 for the biceps was suggested by the data. The threshold criteria were used to track 80 women who had TDC measurements done prior to their breast cancer surgery and then for up to 24 months afterward [61]. In another study, TDC values (forearms and biceps) were obtained in 42 women who were awaiting breast cancer surgery and in 41 healthy women, for a total of 83 inter-arm assessments [78]. A three SD inter-arm threshold ratio of 1.20 and 1.24 for the forearm and biceps was obtained. A test of the suitability of this ratio was done by evaluating 63 women, of whom 32 had BCRL and 31 had breast surgery for cancer but, as of yet, were free of BCRL [79]. Sensitivity and specificity based on inter-arm TDC values were reported as 65% and 94%, respectively. Other studies determined threshold TDC ratios for hands [80]. In this study, 70 women were evaluated, with half <35 and half >50 years of age. An age-independent two SD threshold of 1.23 was reported based on the findings of this study. A study that compared using TDC inter-arm thresholds vs. water displacement found that both were useful, but the TDC method, when used alone, could diagnose lymphedema earlier than the water displacement method [81]. TDC inter-arm ratios have also been used to track the need for reestablishing early-initiated compression to minimize the likelihood of transitioning to chronic lymphedema [82]. It is also noteworthy that TDC measurements have been used to assess the outcomes of treatments such as local cooling [83] and radiotherapy [84].

Breast Lymphedema

Lymphedematous conditions of BCRL do not just involve one arm area, thereby indicating the need for assessments of TDC values in other areas. This was done in a group of women awaiting breast cancer surgery (patients) and a group of healthy women (controls) [49]. Measurements were made at the mid-forearm, mid-biceps, axilla, and lateral thorax at a depth of 2.5 mm. TDC was largest at the axilla (36.4 ± 8.9) and least at the biceps (21.6 ± 3.5). Patient vs. control comparisons showed slightly greater forearm and biceps TDC values for patients but no difference in inter-side TDC values in patients or controls, suggesting that cancer presence itself did not significantly affect TDC values. Additional studies extended these investigations by evaluating 120 women who were awaiting breast cancer surgery [85]. The main aim was to formulate inter-side lateral thorax thresholds. The findings suggested a 2.5 SD thorax-to-thorax TDC threshold ratio of 1.32.

Breast edema or lymphedema can also be present as a complication of breast cancer-related treatment and may be present with or without arm or trunk lymphedema and other conditions [86]. Breast TDC values have been investigated as a way to better characterize the breast edematous condition [87-91]. Based on healthy inter-breast TDC values, an inter-breast diagnostic two SD threshold ratio of 1.40 was reported and applied to 118 women who had received breast cancer surgery and radiotherapy [87]. Based on the 1.40 threshold criterion, it was found that 31.4% of patients had breast edema after surgery, even prior to the end of their radiotherapy. This percentage increased to 62.4% four weeks after the end of radiotherapy. A longer follow-up time of about two years included 65 of these patients, indicating the percentage of these that had inter-breast ratios above the threshold was 28% [88].

The 1.40 threshold criterion was used to investigate a group of 10 women with BCRL [89]. The TDC values of the breast on the affected side were significantly elevated in comparison with the contralateral breast. In addition, the TDC ratio was related to the breast drainage pathway pattern and dermal backflow. TDC methods were also of use in characterizing changes in breast edema in 56 breast cancer-treated patients who had breast edema as determined by the 1.40 threshold [90]. After wearing either a standard or a compression bra for nine months, a reduction in TDC was observed that was correlated with the patient’s perceived feeling of breast heaviness.

Trunk Lymphedema

Another area of interest in patients who have had breast cancer-related surgery is the development of lymphedema in the trunk [92-95]. An interesting observation of this condition has been made from MRI [96]. In this study, a group of 13 women who had BCRL and swelling or subjective symptoms of truncal lymphedema was evaluated. All patients had subjective symptoms and seven had visible swelling, yet the MRI revealed no free water signals on the affected side of the trunk of any patient. Herein, it may be theorized that the swelling and symptoms may be attributable to bound water which is measurable by TDC methods. As noted, these measurements have been taken from healthy subjects, suggesting inter-side thresholds for lymphedema of 1.32. However, they have not yet been correlated with MRI data.

Applications of TDC measurements to lower extremities

Normal Features, Variability, and Thresholds for Lower Extremity TDC Values

**Figure 3:**
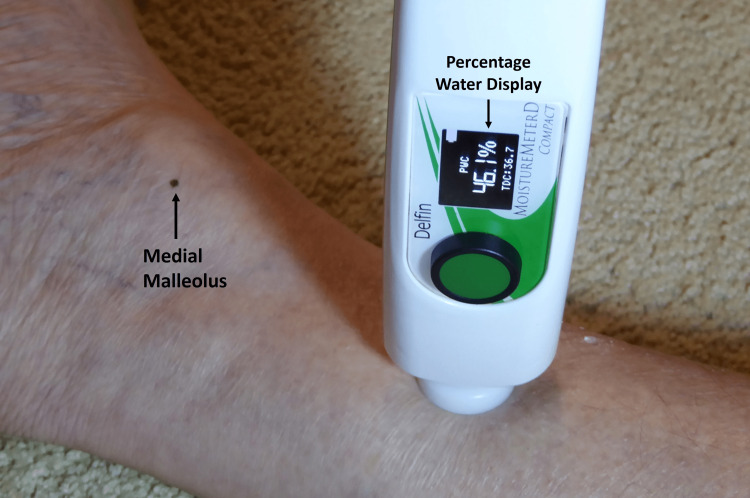
TDC measurement example on the lower extremity A compact version is shown measuring in the gaiter area of the right leg of a patient. Note that the display on the compact device shows the TDC value (36.7) and also the percentage of water (46.1%) at this location. This figure is courtesy of Dr. HN Mayrovitz.

Characteristics of TDC values and features of lower extremities are less studied than those for upper extremities. The locations of lower extremity measurements depend on the purpose of the evaluation. Sites that have been measured include the foot dorsum, the calf or gaiter area (as illustrated in Figure 3) on medial, anterior, and lateral sites, and in some cases the thigh.

In one study designed to investigate site variability, TDC was measured at multiple longitudinal sites on the legs of 18 healthy women at 10, 20, 30, and 40 cm proximal to the medial malleolus on the medial aspect of the leg [97]. In that study, except for the 40 cm location, there was little variability among the TDC values obtained at the other sites. In addition, day-to-day reproducibility via test-retest was reported as excellent. Other measurements of the lower extremities of 30 healthy women showed significant differences between TDC values at the foot, ankle, and calf [98]. TDC values in that study ranged from 37.8 ± 5.5 on the foot to 30.5 ± 3.9 on the lower leg. These workers suggested that TDC values of 35.2 for the ankle and 38.3 for the lower leg are useful as upper normal reference limits in women. The reliability of lower extremity TDC measurements was assessed in a group of 61 healthy subjects (33 female) in whom TDC was measured at multiple leg sites twice, two weeks apart [99]. For TDC measurements, considering all measurement locations, the standard error of the measurement was reported to range from 3.9% to 14.5%. It was suggested that TDC values made near the tendon or bone be carefully interpreted.

Other measurements aimed at characterizing potential differences to be expected among circumferential leg TDC values reported small but statistically significant differences among medial, anterior, and lateral sites when assessed at multiple depths. As a consequence, it was suggested that this fact be considered when evaluating or tracking tissue water changes. The question of differences in TDC values between paired legs was also investigated in a small group of 10 women to determine a potential inter-leg threshold ratio usable for detecting unilateral leg lymphedema [71]. Based on this small sample, an inter-leg threshold of 1.2 was suggested. Because lower extremity lymphedema (LEL) is often bilateral, the search for a method that was not based on inter-leg ratios was sought and proposed [100,101]. This method used the ratio of leg-to-forearm TDC ratios obtained from healthy persons to set a potential lymphedema threshold at 1.35. TDC values above this would suggest the presence of LEL, and changes in this value could be used to track treatment outcomes. Other studies on patients with LEL were consistent with this threshold criterion [102].

Lower Extremity Lymphedema

LEL is a complication of various conditions, including gynecological surgery for various cancers [103-105], as well as for other cancers such as melanoma [106], prostate [107] cancer, and other non-cancer-related conditions [108]. The reported incidence of LEL depends on the method used to determine its presence and ranges from about 35% to 70% [109-111]. The applications of TDC to assessing lower extremity skin features and its potential use for diagnosis and tracking of LEL are more limited than for similar uses in BCRL. In one study, the sensitivity to detect LEL in a group of 106 patients treated mostly for endometrial cancer (N = 52) or ovarian cancer (N =40) was reported to be greater than the standard leg circumference measurement procedure [112]. In that study, bilateral TDC measurements were made on the thigh and calf of the women for a mean follow-up time of about three years. Pitting edema was observed in up to 17.5% of patients, and these patients had a greater measured TDC value than those patients without pitting (35.9 ± 10.4 vs. 27.2 ± 7.5). With respect to quantifying the presence of LEL or tracking its progression, a leg/arm threshold ratio of 1.35 may be useful [104].

Assessing Lymphedema Treatment

Various treatments for LEL are available and include manual lymphatic drainage (MLD) as the most frequently used [113-115]. One study utilized TDC measurements to try to assess the utility of a single MLD treatment session and was able to use pre- and post-treatment TDC measurements to achieve this [116]. Other treatment modalities for LEL that have used TDC measurements to assess outcomes include laser therapy [117] and pneumatic compression [118]. In addition, the use of TDC measurements has been evaluated as a method to distinguish LEL from lipoedema [119].

Applications of TDC measurements for various conditions

Diabetic Skin Assessment

The potential linkages between diabetes mellitus (DM) and potential skin problems are well documented [120-122]. Because of this connection, some studies have attempted to use TDC measurements to assess certain features of skin in persons with DM. To investigate skin water status, TDC was measured in 36 persons (18 with DM) to depths of 0.5, 1.5, and 2.5 mm at the foot dorsum and forearm bilaterally [123]. It was reported that all persons with DM had higher foot dorsum TDC values than those without DM, whereas values at the forearm did not significantly differ between groups. The TDC values of the DM group, when measured to a depth of 2.5 mm, were about 15% greater. This finding suggested that unrecognized edema was present. Subsequent research focused on the potential link between HbA1c values and skin water as measured using the TDC method [124]. In that study, TDC was measured in 50 patients with DM at depths of 0.5, 1.5, 2.5, and 5.0 mm at the foot dorsum, calf, and forearm. Though there was a significant increase in foot-to-arm TDC values, there was no significant relationship found between the absolute TDC value and HbA1c or the patient’s fasting glucose level.

Dehydration Status

Because TDC values are strongly dependent on skin tissue water, the use of this method to assess body water status has been evaluated in several studies. The possibility of tracking fluid status using TDC measurements was evaluated by measuring TDC on the forearm, thigh, and abdomen in 29 patients prior to their cardiac surgery and for some days afterward [25]. By weighing the patient, a significant correlation between TDC values and weight was reported for weight gain from baseline to the second post-operative day (r = 0.600, p<0.01) but not for a weight loss interval from days two to four. These data leave the question of tracking dehydration status unresolved. However, more recent information derived from 10 healthy exercising subjects indicates a tracking of hydration status via TDC values [125]. Other studies, in which the relationship between total body water percentage and measured TDC values was investigated, were also consistent with TDC being a possible indicator of body fluid status [62].

Wound Feature Assessment

Several workers have addressed the issue of using TDC measurements in conjunction with wound assessments. An early study compared wounded skin with normal skin and reported that the wounded skin had about a 12% higher value [126]. Subsequently, the open-ended coaxial line method was used to evaluate the skin injury produced by irradiated breast skin [127]. A two-year follow-up of 21 patients indicated as high as a 39% reduction in TDC that correlated with the presence of subcutaneous fibrosis. Pressure ulcers in patients with spinal cord injury were evaluated using this method to a depth of 1.5 mm, in which peri-wound skin areas were compared to non-injured skin [128]. The values obtained were among the first to report peri-wound values in this type of setting and condition. Further research into the use of TDC-related measurements indicated their potential use to detect early phases of pressure ulcer development at the sacrum [129] and heel [130]. The TDC method has also been suggested as a useful approach for characterizing and tracking changes in lymphedema associated with head and neck cancer [131].

## Conclusions

The findings demonstrate multiple research and medical uses and applications of TDC measurements, focusing on detecting and quantifying localized edema and lymphedema in multiple target sites. These include the upper and lower extremities, breasts, and trunk as regions involved in medical conditions causing lymphedema. In addition, the findings suggest that TDC evaluations are a convenient, non-invasive method to study and evaluate other conditions impacting skin, including DM and skin wounds or ulcers. Its ability to detect aspects of tissue changes simply and rapidly at almost any anatomical location makes it a useful tool for investigating multiple dermatological conditions and their treatment as future applications of this method.

## References

[1] Mayrovitz HN (2007). Assessing local tissue edema in postmastectomy lymphedema. Lymphology.

[2] Pauly H, Schwan HP (1966). Dielectric properties and ion mobility in erythrocytes. Biophys J.

[3] Pennock BE, Schwan HP (1969). Further observations on the electrical properties of hemoglobin-bound water. J Phys Chem.

[4] Schwan HP, Foster KR (1977). Microwave dielectric properties of tissue. Some comments on the rotational mobility of tissue water. Biophys J.

[5] Foster KR, Schwan HP (1989). Dielectric properties of tissues and biological materials: a critical review. Crit Rev Biomed Eng.

[6] Cole KS (1928). Electric impedance of suspensions of Arbacia eggs. J Gen Physiol.

[7] CO KS (1962). The advance of electrical models for cells and axons. Biophys J.

[8] Stuchly MA, Athey TW, Stuchly SS, Samaras GM, Taylor G (1981). Dielectric properties of animal tissues in vivo at frequencies 10 MHz--1 GHz. Bioelectromagnetics.

[9] Stuchly MA, Athey TW, Samaras GM, Taylor GE (1982). Measurement of radio frequency permittivity of biological tissues with an open-ended coaxial line: part II - experimental results. IEEE Trans Microw Theory Tech.

[10] Stuchly MA, Kraszewski A, Stuchly SS, Smith AM (1982). Dielectric properties of animal tissues in vivo at radio and microwave frequencies: comparison between species. Phys Med Biol.

[11] Smith SR, Foster KR (1985). Dielectric properties of low-water-content tissues. Phys Med Biol.

[12] Gabriel S, Lau RW, Gabriel C (1996). The dielectric properties of biological tissues: II. Measurements in the frequency range 10 Hz to 20 GHz. Phys Med Biol.

[13] Grant JP, Clarke RN, Symm GT, Spyrou NM (1988). In vivo dielectric properties of human skin from 50 MHz to 2.0 GHz. Phys Med Biol.

[14] Alanen E, Lahtinen T, Nuutinen J (1998). Variational formulation of open-ended coaxial line in contact with layered biological medium. IEEE Trans Biomed Eng.

[15] Alanen E, Lahtinen T, Nuutinen J (1998). Measurement of dielectric properties of subcutaneous fat with open-ended coaxial sensors. Phys Med Biol.

[16] Alanen E, Lahtinen T, Nuutinen J (1999). Penetration of electromagnetic fields of an open-ended coaxial probe between 1 MHz and 1 GHz in dielectric skin measurements. Phys Med Biol.

[17] Nuutinen J, Ikäheimo R, Lahtinen T (2004). Validation of a new dielectric device to assess changes of tissue water in skin and subcutaneous fat. Physiol Meas.

[18] La Gioia A, Porter E, Merunka I, Shahzad A, Salahuddin S, Jones M, O'Halloran M (2018). Open-ended coaxial probe technique for dielectric measurement of biological tissues: challenges and common practices. Diagnostics (Basel).

[19] Mayrovitz HN, Weingrad DN, Brlit F, Lopez LB, Desfor R (2015). Tissue dielectric constant (tdc) as an index of localized arm skin water: differences between measuring probes and genders. Lymphology.

[20] Etoz S, Brace CL (2019). Development of water content dependent tissue dielectric property models. IEEE J Electromagn RF Microw Med Biol.

[21] Kao HP, Cardoso ER, Shwedyk E (1990). Measurement of canine cerebral oedema using time domain reflectometry. Acta Neurochir Suppl (Wien).

[22] Kramer GG, Cardoso ER, Shwedyk E (1992). Dielectric measurement of cerebral water content using a Network Analyzer. Neurol Res.

[23] Kao HP, Cardoso ER, Shwedyk E (1999). Correlation of permittivity and water content during cerebral edema. IEEE Trans Biomed Eng.

[24] Miura N, Shioya S, Kurita D, Shigematsu T, Mashimo S (1999). Time domain reflectometry: measurement of free water in normal lung and pulmonary edema. Am J Physiol.

[25] Petäjä L, Nuutinen J, Uusaro A, Lahtinen T, Ruokonen E (2003). Dielectric constant of skin and subcutaneous fat to assess fluid changes after cardiac surgery. Physiol Meas.

[26] Rochlin DH, Barrio AV, McLaughlin S (2023). Feasibility and clinical utility of prediction models for breast cancer-related lymphedema incorporating racial differences in disease incidence. JAMA Surg.

[27] Ganju RG, Savvides G, Korentager S (2019). Incidence of breast lymphedema and predictors of its development in patients receiving whole breast radiation therapy after breast-conservation surgery. Lymphology.

[28] Koelmeyer LA, Borotkanics RJ, Alcorso J (2019). Early surveillance is associated with less incidence and severity of breast cancer-related lymphedema compared with a traditional referral model of care. Cancer.

[29] Zou L, Liu FH, Shen PP (2018). The incidence and risk factors of related lymphedema for breast cancer survivors post-operation: a 2-year follow-up prospective cohort study. Breast Cancer.

[30] Ribeiro Pereira AC, Koifman RJ, Bergmann A (2017). Incidence and risk factors of lymphedema after breast cancer treatment: 10 years of follow-up. Breast.

[31] Esmer M, Schingale FJ (2023). Effect of physical therapy on circumference measurement and extremity volume in patients suffering from lipedema with secondary lymphedema. Lymphat Res Biol.

[32] Tokumoto H, Akita S, Kubota Y, Mitsukawa N (2022). Relationship between the circumference difference and findings of indocyanine green lymphography in breast cancer-related lymphedema. Ann Plast Surg.

[33] Furlan C, Matheus CN, Jales RM, Derchain SF, Bennini JR Jr, Sarian LO (2021). Longitudinal, long-term comparison of single- versus multipoint upper limb circumference periodical measurements as a tool to predict persistent lymphedema in women treated surgically for breast cancer: an optimized strategy to early diagnose lymphed. Ann Surg Oncol.

[34] Wang H, Shen L, Liu T, Shao P, Dylke ES, Jia J, Kilbreath SL (2017). Circumference-based criteria for detection of secondary arm lymphedema for Chinese women. Lymphat Res Biol.

[35] Mastick J, Smoot BJ, Paul SM (2022). Assessment of arm volume using a tape measure versus a 3D optical scanner in survivors with breast cancer-related lymphedema. Lymphat Res Biol.

[36] Kim N, Kim H, Hwang JH (2021). Longitudinal impact of postmastectomy radiotherapy on arm lymphedema in patients with breast cancer: an analysis of serial changes in arm volume measured by infrared optoelectronic volumetry. Radiother Oncol.

[37] Specht MC, Miller CL, Russell TA (2013). Defining a threshold for intervention in breast cancer-related lymphedema: what level of arm volume increase predicts progression?. Breast Cancer Res Treat.

[38] Meijer RS, Rietman JS, Geertzen JH, Bosmans JC, Dijkstra PU (2004). Validity and intra- and interobserver reliability of an indirect volume measurements in patients with upper extremity lymphedema. Lymphology.

[39] Sander AP, Hajer NM, Hemenway K, Miller AC (2002). Upper-extremity volume measurements in women with lymphedema: a comparison of measurements obtained via water displacement with geometrically determined volume. Phys Ther.

[40] Soran A, Bengur FB, Rodriguez W, Chroneos MZ, Sezgin E (2023). Early detection of breast cancer-related lymphedema: accuracy of indocyanine green lymphography compared with bioimpedance spectroscopy and subclinical lymphedema symptoms. Lymphat Res Biol.

[41] Kaufman DI, Shah C, Vicini FA, Rizzi M (2017). Utilization of bioimpedance spectroscopy in the prevention of chronic breast cancer-related lymphedema. Breast Cancer Res Treat.

[42] Rockson SG (2015). Detecting lymphedema: Bioimpedance spectroscopy and the tissue dielectric constant. Lymphat Res Biol.

[43] Shah C, Vicini F, Beitsch P, Laidley A, Anglin B, Lyden M (2013). The use of bioimpedance spectroscopy to monitor therapeutic intervention in patients treated for breast cancer related lymphedema. Lymphology.

[44] Ward LC, Czerniec S, Kilbreath SL (2009). Operational equivalence of bioimpedance indices and perometry for the assessment of unilateral arm lymphedema. Lymphat Res Biol.

[45] Toshima M, Morino Y (2022). Water distribution changes in complex decongestive treatment for leg lymphedema: quantitative evaluation by direct segmental multi-frequency bioimpedance analysis. Ann Vasc Dis.

[46] Moseley A, Piller N, Carati C (2002). Combined opto-electronic perometry and bioimpedance to measure objectively the effectiveness of a new treatment intervention for chronic secondary leg lymphedema. Lymphology.

[47] Mayrovitz HN, Sims N, Macdonald J (2000). Assessment of limb volume by manual and automated methods in patients with limb edema or lymphedema. Adv Skin Wound Care.

[48] Kaulesar Sukul DM, den Hoed PT, Johannes EJ, van Dolder R, Benda E (1993). Direct and indirect methods for the quantification of leg volume: comparison between water displacement volumetry, the disk model method and the frustum sign model method, using the correlation coefficient and the limits of agreement. J Biomed Eng.

[49] Mayrovitz HN, Davey S, Shapiro E (2008). Local tissue water assessed by tissue dielectric constant: anatomical site and depth dependence in women prior to breast cancer treatment-related surgery. Clin Physiol Funct Imaging.

[50] Maenhout G, Markovic T, Ocket I, Nauwelaers B (2020). Effect of open-ended coaxial probe-to-tissue contact pressure on dielectric measurements. Sensors (Basel).

[51] Mayrovitz HN, Davey S, Shapiro E (2008). Local tissue water changes assessed by tissue dielectric constant: single measurements versus averaging of multiple measurements. Lymphology.

[52] Mayrovitz HN, Davey S, Shapiro E (2009). Suitability of single tissue dielectric constant measurements to assess local tissue water in normal and lymphedematous skin. Clin Physiol Funct Imaging.

[53] Mayrovitz HN (2019). Assessing upper and lower extremities via tissue dielectric constant: suitability of single versus multiple measurements averaged. Lymphat Res Biol.

[54] Mayrovitz HN, Luis M (2010). Spatial variations in forearm skin tissue dielectric constant. Skin Res Technol.

[55] Mayrovitz HN, Bernal M, Brlit F, Desfor R (2013). Biophysical measures of skin tissue water: variations within and among anatomical sites and correlations between measures. Skin Res Technol.

[56] Koehler LA, Mayrovitz HN (2019). Spatial and temporal variability of upper extremity edema measures after breast cancer surgery. Lymphat Res Biol.

[57] Mayrovitz HN, Fasen M, Spagna P, Wong J (2018). Role of handedness on forearm skin tissue dielectric constant (TDC) in relation to detection of early-stage breast cancer-related lymphedema. Clin Physiol Funct Imaging.

[58] Mayrovitz HN, Arzanova E, Somarriba S, Eisa S (2019). Factors affecting interpretation of tissue dielectric constant (tdc) in assessing breast cancer treatment related lymphedema (BCRL). Lymphology.

[59] Mayrovitz HN (2022). Circumferential and depth variations in tissue dielectric constant values as indices of lower leg localized skin water. Cureus.

[60] Mayrovitz HN, Grammenos A, Corbitt K, Bartos S (2016). Young adult gender differences in forearm skin-to-fat tissue dielectric constant values measured at 300 MHz. Skin Res Technol.

[61] Mayrovitz HN, Weingrad DN, Lopez L (2015). Patterns of temporal changes in tissue dielectric constant as indices of localized skin water changes in women treated for breast cancer: a pilot study. Lymphat Res Biol.

[62] Mayrovitz HN, Arzanova E, Somarriba S, Eisa S (2019). Factors affecting interpretation of tissue dielectric constant (tdc) in assessing breast cancer treatment related lymphedema (bcrl). Lymphology.

[63] Mayrovitz HN, Forbes J, Vemuri A, Krolick K, Rubin S (2020). Skin tissue dielectric constant in women with high body fat content. Skin Res Technol.

[64] Mayrovitz HN (2010). Local tissue water assessed by measuring forearm skin dielectric constant: dependence on measurement depth, age and body mass index. Skin Res Technol.

[65] Mayrovitz HN, Singh A, Akolkar S (2016). Age-related differences in tissue dielectric constant values of female forearm skin measured noninvasively at 300 MHz. Skin Res Technol.

[66] Mayrovitz HN, Grammenos A, Corbitt K, Bartos S (2017). Age-related changes in male forearm skin-to-fat tissue dielectric constant at 300 MHz. Clin Physiol Funct Imaging.

[67] Mayrovitz HN, Wong J, Fasen M (2018). Age and hydration dependence of jowl and forearm skin firmness in young and mature women. J Cosmet Dermatol.

[68] Mayrovitz HN, Bernal M, Carson S (2012). Gender differences in facial skin dielectric constant measured at 300 MHz. Skin Res Technol.

[69] Mayrovitz HN, Mahtani SA, Pitts E, Michaelos L (2017). Race-related differences in tissue dielectric constant measured noninvasively at 300 MHz in male and female skin at multiple sites and depths. Skin Res Technol.

[70] Mayrovitz HN (2017). Diurnal changes in local skin water assessed via tissue dielectric constant at 300 MHz. Biomed Phys Eng Express.

[71] Mayrovitz HN (2022). Tissue dielectric constant of the lower leg as an index of skin water: temporal variations. Cureus.

[72] Mayrovitz HN, Mikulka A, Woody D (2019). Minimum detectable changes associated with tissue dielectric constant measurements as applicable to assessing lymphedema status. Lymphat Res Biol.

[73] De Vrieze T, Gebruers N, Nevelsteen I (2020). Reliability of the moisturemeterd compact device and the pitting test to evaluate local tissue water in subjects with breast cancer-related lymphedema. Lymphat Res Biol.

[74] Mayrovitz HN, Weingrad DN, Davey S (2009). Local tissue water in at-risk and contralateral forearms of women with and without breast cancer treatment-related lymphedema. Lymphat Res Biol.

[75] Mayrovitz HN, Weingrad DN, Davey S (2014). Tissue dielectric constant (TDC) measurements as a means of characterizing localized tissue water in arms of women with and without breast cancer treatment related lymphedema. Lymphology.

[76] Lahtinen T, Seppälä J, Viren T, Johansson K (2015). Experimental and analytical comparisons of tissue dielectric constant (TDC) and bioimpedance spectroscopy (BIS) in assessment of early arm lymphedema in breast cancer patients after axillary surgery and radiotherapy. Lymphat Res Biol.

[77] Mayrovitz HN, Weingrad DN, Lopez L (2015). Assessing localized skin-to-fat water in arms of women with breast cancer via tissue dielectric constant measurements in pre- and post-surgery patients. Ann Surg Oncol.

[78] Mayrovitz HN, Weingrad DN, Lopez L (2016). Tissue dielectric constant (TDC) as an index of skin water in women with and without breast cancer: upper limb assessment via a self-contained compact measurement device. Lymphology.

[79] Bakar Y, Tuğral A, Üyetürk Ü (2018). Measurement of local tissue water in patients with breast cancer-related lymphedema. Lymphat Res Biol.

[80] Mayrovitz HN, Arzanova E, Somarriba S, Eisa S (2018). Reference values for assessing localized hand lymphedema using Interhand tissue dielectric constant ratios. Lymphat Res Biol.

[81] Karlsson K, Nilsson-Wikmar L, Brogårdh C, Johansson K (2020). Palpation of increased skin and subcutaneous thickness, tissue dielectric constant, and water displacement method for diagnosis of early mild arm lymphedema. Lymphat Res Biol.

[82] Johansson K, Blom K, Nilsson-Wikmar L, Brogårdh C (2023). Early intervention with a compression sleeve in mild breast cancer-related arm lymphedema: a 12-month prospective observational study. Cancers (Basel).

[83] Mayrovitz HN, Yzer JA (2017). Local skin cooling as an aid to the management of patients with breast cancer related lymphedema and fibrosis of the arm or breast. Lymphology.

[84] Tuğral A, Akyol M, Bakar Y (2023). The effect of adjuvant radiotherapy on skin biophysical properties in patients with breast cancer at risk for breast lymphedema: a prospective study. Clin Physiol Funct Imaging.

[85] Mayrovitz HN, Weingrad DN (2018). Tissue dielectric constant ratios as a method to characterize truncal lymphedema. Lymphology.

[86] Gupta SS, Mayrovitz HN (2022). The breast edema Enigma: features, diagnosis, treatment, and recommendations. Cureus.

[87] Johansson K, Lathinen T, Björk-Eriksson T (2014). Breast edema following conserving surgery and radiotherapy. Eur J Lymphology.

[88] Johansson K, Darkeh MH, Lahtinen T, Bjork-Eriksson T, Axelssqn R (2015). Two-year follow-up of temporal changes of breast edema after breast cancer treatment with surgery and radiation evaluated by tissue dielectric constant (TDC). Eur J Lymphology Relat Probl.

[89] Heydon-White A, Suami H, Boyages J, Koelmeyer L, Peebles KC (2020). Assessing breast lymphoedema following breast cancer treatment using indocyanine green lymphography. Breast Cancer Res Treat.

[90] Johansson K, Jönsson C, Björk-Eriksson T (2020). Compression treatment of breast edema: a randomized controlled pilot study. Lymphat Res Biol.

[91] Mayrovitz HN, Somarriba C, Weingrad DN (2022). Breast tissue dielectric constant as a potential breast edema assessment parameter. Lymphat Res Biol.

[92] Koehler LA, Mayrovitz HN (2020). Tissue dielectric constant measures in women with and without clinical trunk lymphedema following breast cancer surgery: a 78-week longitudinal study. Phys Ther.

[93] Mayrovitz HN (2021). Noninvasive measurements of breast cancer-related lymphedema. Cureus.

[94] Ren Y, Kebede MA, Ogunleye AA (2022). Burden of lymphedema in long-term breast cancer survivors by race and age. Cancer.

[95] Ulman J Dr, Serrant L Professor, Dunham M Dr, Probst H Professor (2023). Exploring women's experiences of breast or trunk lymphoedema following treatment for breast cancer. J Psychosoc Oncol.

[96] Hisano F, Niwa S, Nakanishi K (2021). The correlation between fluid distribution and swelling or subjective symptoms of the trunk in lymphedema patients: a preliminary observational study. Lymphat Res Biol.

[97] Ehmann S, Mayrovitz HN (2023). Variation in leg tissue dielectric constant values of healthy young adult females with and without compression bandaging. Cureus.

[98] Jensen MR, Birkballe S, Nørregaard S, Karlsmark T (2012). Validity and interobserver agreement of lower extremity local tissue water measurements in healthy women using tissue dielectric constant. Clin Physiol Funct Imaging.

[99] Jönsson C, Bjurberg M, Brogårdh C, Johansson K (2020). Test-retest reliability of volume and local tissue water measurements in lower limbs of healthy women and men. Lymphat Res Biol.

[100] Mayrovitz HN, Alvarez A, Labra M, Mikulka A, Woody D (2019). Possible applications of normative lower to upper limb ratios of tissue dielectric constant to lower extremity edema. Int Angiol.

[101] Mayrovitz HN (2019). Assessing lower extremity lymphedema using upper and lower extremity tissue dielectric constant ratios: method and normal reference values. Lymphat Res Biol.

[102] Mayrovitz HN, Shams E, Astudillo A, Jain A (2023). Tissue dielectric constant and skin stiffness relationships in lower extremity lymphedema. Lymphat Res Biol.

[103] Armbrust R, Auletta V, Cichon G, Vercellino G, Yost K, Sehouli J (2023). Lymphedema after pelvic and para-aortic lymphadenectomy-results of a systematic evaluation in patients with cervical and endometrial carcinoma. Arch Gynecol Obstet.

[104] Jung SG, Im SH, Kim M (2022). The association between the number of retrieved pelvic lymph nodes and ipsilateral lower limb lymphedema in patients with gynecologic cancer. J Invest Surg.

[105] Lee J, Byun HK, Im SH, Son WJ, Roh YH, Kim YB (2023). Risk factors for lower extremity lymphedema after surgery in cervical and endometrial cancer. J Gynecol Oncol.

[106] Chae Woon P, Kim I, Kim JH, Hwang JH (2023). Association of clinical manifestations of secondary lymphedema and lymph node dissection sites in the lower extremities of patients with melanoma. Acta Oncol.

[107] Neuberger M, Schmidt L, Wessels F (2022). Onset and burden of lower limb lymphedema after radical prostatectomy: a cross-sectional study. Support Care Cancer.

[108] Dean SM, Valenti E, Hock K, Leffler J, Compston A, Abraham WT (2020). The clinical characteristics of lower extremity lymphedema in 440 patients. J Vasc Surg Venous Lymphat Disord.

[109] Abu-Rustum NR, Alektiar K, Iasonos A (2006). The incidence of symptomatic lower-extremity lymphedema following treatment of uterine corpus malignancies: a 12-year experience at Memorial Sloan-Kettering Cancer Center. Gynecol Oncol.

[110] Carlson JW, Kauderer J, Hutson A (2020). GOG 244-The lymphedema and gynecologic cancer (LEG) study: Incidence and risk factors in newly diagnosed patients. Gynecol Oncol.

[111] Ki EY, Park JS, Lee KH, Hur SY (2016). Incidence and risk factors of lower extremity lymphedema after gynecologic surgery in ovarian cancer. Int J Gynecol Cancer.

[112] Tuppurainen MT, Lahtinen T, Pyykönen J, Komulainen M, Nuutinen J, Anttila M (2011). Lymphoedema of lower limb following treatment for gynaecological cancer. Int J Gynecol Cancer.

[113] Liu F, Liu NF, Wang L, Chen J, Han L, Yu Z, Sun D (2021). Treatment of secondary lower limb lymphedema after gynecologic cancer with complex decongestive therapy. Lymphology.

[114] Wang X, Ding Y, Cai HY, You J, Fan FQ, Cai ZF, An P (2020). Effectiveness of modified complex decongestive physiotherapy for preventing lower extremity lymphedema after radical surgery for cervical cancer: a randomized controlled trial. Int J Gynecol Cancer.

[115] Kendrová L, Mikuľáková W, Urbanová K, Andraščíková Š, Žultáková S, Takáč P, Peresta Y (2020). Comprehensive decongestive therapy as a treatment for secondary lymphedema of the lower extremity and quality of life of women after gynecological cancer surgery. Med Sci Monit.

[116] Mayrovitz HN, Davey S, Shapiro E (2008). Localized tissue water changes accompanying one manual lymphatic drainage (MLD) therapy session assessed by changes in tissue dielectric constant in patients with lower extremity lymphedema. Lymphology.

[117] Mayrovitz HN, Davey S (2011). Changes in tissue water and indentation resistance of lymphedematous limbs accompanying low level laser therapy (lllt) of fibrotic skin. Lymphology.

[118] Zaleska MT, Olszewski WL (2019). The effectiveness of intermittent pneumatic compression in therapy of lymphedema of lower limbs: methods of evaluation and results. Lymphat Res Biol.

[119] Birkballe S, Jensen MR, Noerregaard S, Gottrup F, Karlsmark T (2014). Can tissue dielectric constant measurement aid in differentiating lymphoedema from lipoedema in women with swollen legs?. Br J Dermatol.

[120] de Macedo GM, Nunes S, Barreto T (2016). Skin disorders in diabetes mellitus: an epidemiology and physiopathology review. Diabetol Metab Syndr.

[121] Makrantonaki E, Jiang D, Hossini AM, Nikolakis G, Wlaschek M, Scharffetter-Kochanek K, Zouboulis CC (2016). Diabetes mellitus and the skin. Rev Endocr Metab Disord.

[122] Mendes AL, Miot HA, Haddad V Junior (2017). Diabetes mellitus and the skin. An Bras Dermatol.

[123] Mayrovitz HN, McClymont A, Pandya N (2013). Skin tissue water assessed via tissue dielectric constant measurements in persons with and without diabetes mellitus. Diabetes Technol Ther.

[124] Mayrovitz HN, Volosko I, Sarkar B, Pandya N (2017). Arm, leg, and foot skin water in persons with diabetes mellitus (DM) in relation to HbA1c assessed by tissue dielectric constant (TDC) technology measured at 300 MHz. J Diabetes Sci Technol.

[125] Eda N, Nakamura N, Inai Y, Sun Z, Sone R, Watanabe K, Akama T (2023). Changes in the skin characteristics associated with dehydration and rehydration. Eur J Sport Sci.

[126] Gabriel C, Bentall RH, Grant EH (1987). Comparison of the dielectric properties of normal and wounded human skin material. Bioelectromagnetics.

[127] Nuutinen J, Lahtinen T, Turunen M, Alanen E, Tenhunen M, Usenius T, Kolle R (1998). A dielectric method for measuring early and late reactions in irradiated human skin. Radiother Oncol.

[128] Harrow JJ, Mayrovitz HN (2014). Subepidermal moisture surrounding pressure ulcers in persons with a spinal cord injury: a pilot study. J Spinal Cord Med.

[129] Bates-Jensen BM, McCreath HE, Patlan A (2017). Subepidermal moisture detection of pressure induced tissue damage on the trunk: the pressure ulcer detection study outcomes. Wound Repair Regen.

[130] Bates-Jensen BM, McCreath HE, Nakagami G, Patlan A (2018). Subepidermal moisture detection of heel pressure injury: the pressure ulcer detection study outcomes. Int Wound J.

[131] Mayrovitz HN, Patel A, Kavadi R, Khan Z, Bartolone S (2021). An approach toward assessing head-and-neck lymphedema using tissue dielectric constant ratios: method and normal reference values. Lymphat Res Biol.

